# Study on the application of preoperative three-dimensional CT angiography of perigastric arteries in laparoscopic radical gastrectomy

**DOI:** 10.1038/s41598-022-09584-0

**Published:** 2022-04-11

**Authors:** Peng Liu, Meng Wei, Danping Sun, Xin Zhong, Yize Liang, Jun Ouyang, Yuan Zhang, Wenbin Yu

**Affiliations:** 1 Department of Gastrointestinal Surgery, Qilu Hospital of Shandong University, No. 107, Wenhuaxi Road, Jinan, 250012 Shandong Province China; 2 Clinical Epidemiology Unit, Qilu Hospital of Shandong University, Jinan, China

**Keywords:** Anatomy, Gastroenterology, Oncology

## Abstract

To investigate the clinical value and significance of preoperative three-dimensional computerized tomography angiography (CTA) in laparoscopic radical gastrectomy for gastric cancer. The clinical data were analyzed retrospectively from 214 gastric cancer patients. We grouped according to whether to perform CTA, and we compared and analyzed the difference of the data between the two groups. The perigastric arteries were classified according to CTA images of patients in the CTA group. The celiac trunk was classified according to Adachi classification: Type I (118/125, 94.4%), Type II (3/125, 2.4%), Type III (0/125, 0%), Type IV (1/125, 0.8%), Type V (2/125, 1.6%), Type VI (1/125, 0.8%). Hepatic artery classification was performed according to Hiatt classification: Type I (102/125, 81.6%), Type II (9/125, 7.2%), Type III (6/125, 4.8%), Type IV (2/125, 1.6%), Type V (3/125, 2.4%), Type VI (0, 0%), Others (3/125, 2.4%). And this study combined vascular anatomy and surgical risk to establish a new splenic artery classification model. In comparison, the operation time, first exhaust time, and estimated blood loss in the CTA group were significantly lower than those in the non-CTA group. In addition, the blood loss in the CTA group combined with ICG (Indocyanine Green) labeled fluorescence laparoscopy was significantly less than that in the group without ICG labeled. Preoperative CTA could objectively evaluate patients' vascular route and variation and then help us avoid or decrease the risk of vascular injury and bleeding. When combined with ICG labeled fluorescence laparoscopy, it could further reduce the risk of iatrogenic injury during the operation and improve postoperative recovery.

## Introduction

In recent years, since the high incidence and mortality of gastric cancer, it threatened human health increasingly^1^. Although many therapies such as chemotherapy, immunotherapy, and targeted therapy had achieved beneficial results in treating gastric cancer, a surgical operation is still the most effective treatment for gastric cancer^2^. In 1994, Kitano et al.^3^ first reported laparoscopic-assisted radical gastrectomy for gastric cancer. Subsequently, Goh et al.^4^ performed laparoscopic D2 radical gastrectomy for advanced gastric cancer for the first time in 1997 and achieved satisfying short-term efficacy. Compared to open surgery, laparoscopic surgery has the advantages of a small incision, good surgical field, less tumor extrusion, and rapid postoperative recovery. With the rapid development of laparoscopic equipment and technique^5^, laparoscopic surgery gradually became the first choice for surgeons and patients.

Lymph node dissection has a significant impact on the pathological staging of patients after surgery. The American Joint Committee on Cancer Staging manual recommends that at least 16 lymph nodes be checked; collecting > 16 nodes can prevent gastric cancer patients from being classified as N3b^6^. The lymph nodes mainly distributed along blood vessels determine the importance of dealing with blood vessels for laparoscopic radical gastric cancer surgery, so the precise positioning of blood vessels is critical. However, compared with traditional open surgery, the surgeon cannot directly sense the blood vessels in the abdominal cavity with his hands during laparoscopic surgery, which makes the positioning of the blood vessels in operation not precise enough. In addition, there are many variations in the blood vessels around the stomach, and the risk of an accidental injury is high. This limitation has always been an urgent problem in laparoscopic radical gastric cancer surgery. Before surgery, the traditional CT scan, gastroscopy, and upper gastrointestinal angiography cannot clearly show the course of perigastric arteries. The discovery of vascular variation makes preoperative assessment and surgical risk prediction have certain limitations. However, CT angiography (CTA) can effectively make up for the shortcomings of the inspection mentioned above methods. CTA is extensive use of CT scanning and three-dimensional reconstruction technology, which could identify the course of blood vessels more clearly. Some studies have shown that CTA is superior to traditional angiography in diagnostic accuracy, safety, patient compliance, and examination time^7^. Based on CT, 3D images constructed with the help of CTA could simulate the three-dimensional state of perigastric blood vessels. That could be helpful for surgeons to make surgical plans and guide intraoperative operations, reduce collateral injuries and avoid operation-related complications^8^. We analyzed the preoperative CTA results of the experimental group to understand the perigastric vascular course and variation. We compared the short-term clinical outcomes with the non-CTA group, aiming to evaluate the clinical application value of preoperative CTA in laparoscopic radical gastrectomy for gastric cancer.

## Materials and methods

### Clinical data

**Figure 1:**
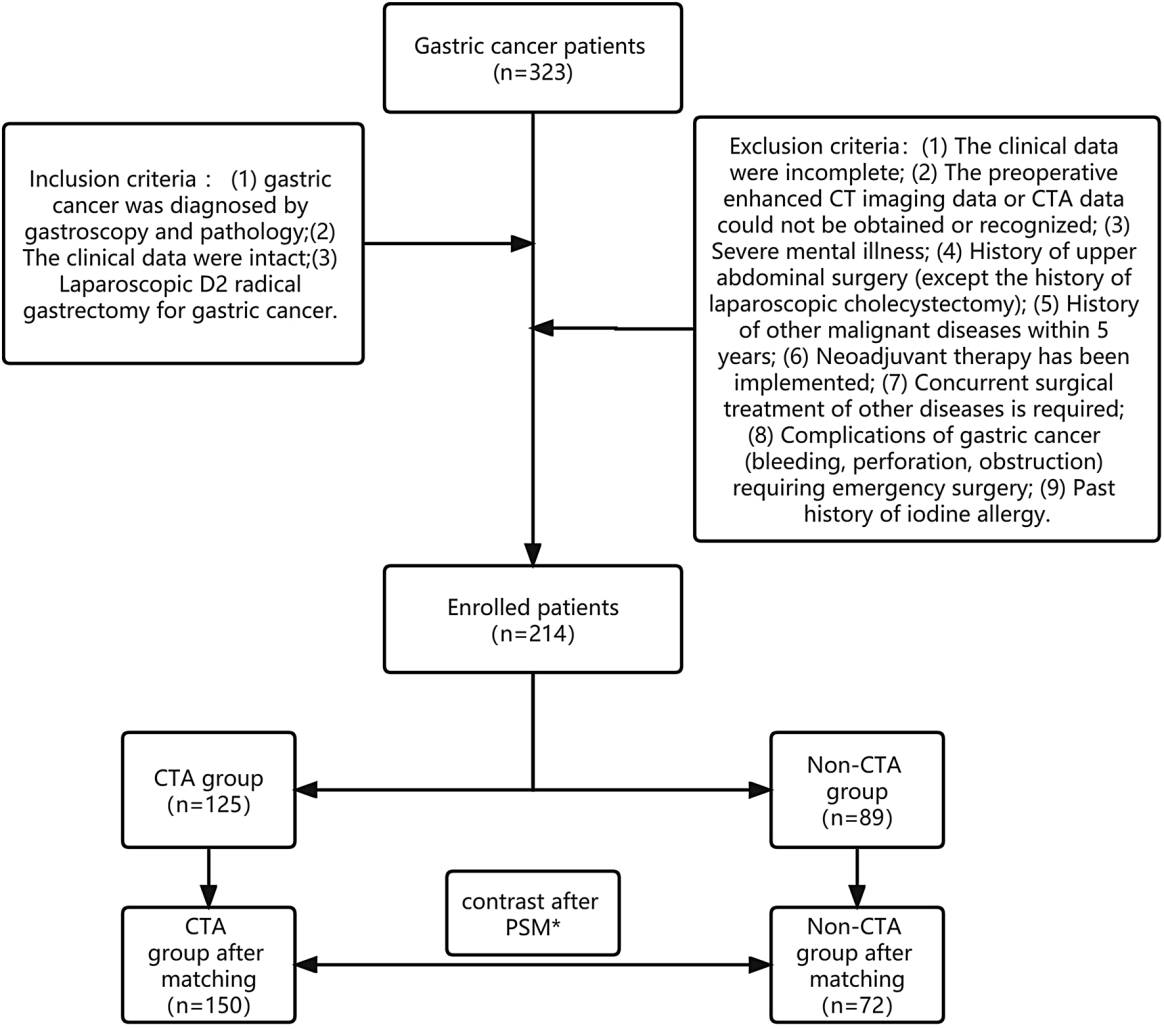
Research flow chart of gastric cancer patients. *Propensity Score Matching.

This study prospectively collected and retrospectively analyzed the clinical data of 214 patients who underwent laparoscopic D2 radical resection of gastric cancer from August 2018 to April 2021 in the Department of Gastrointestinal Surgery, Qilu Hospital Shandong University. In the CTA group, 125 patients underwent a traditional enhanced CT scan and CTA examination of the celiac trunk artery before operation. And 89 patients only examined by conventional enhanced CT scan were grouped in the non-CTA group. Inclusion criteria were (1) gastric cancer was diagnosed by gastroscopy and pathology; (2)The clinical data were intact; (3) Laparoscopic D2 radical gastrectomy for gastric cancer. Exclusion criteria were (1) The clinical data were incomplete; (2) The preoperative enhanced CT imaging data or CTA data could not be obtained or recognized; (3) Severe mental illness; (4) History of upper abdominal surgery (except the history of laparoscopic cholecystectomy); (5) History of other malignant diseases within five years; (6) Neoadjuvant therapy has been implemented; (7) Concurrent surgical treatment of other diseases is required; (8) Complications of gastric cancer (bleeding, perforation, obstruction) requiring emergency surgery; (9) History of an iodine allergy (Fig. 1).

### Anatomical study

Two senior radiologists and one gastroenterologist reviewed the preoperative CTA results of 125 patients to distinguish the origin and course of perigastric artery: abdominal aorta (AA), celiac trunk, common hepatic artery (CHA), splenic artery (SA), left gastric artery (LGA), proper hepatic artery (PHA), right hepatic artery (RHA), left hepatic artery (LHA), abnormal left hepatic artery (ALHA, alternative LHA or accessory LHA), abnormal right hepatic artery (ARHA, alternative RHA or accessory RHA), right gastric artery (RGA). The perigastric arteries were classified according to the specific classification method below.

The classification of the celiac trunk and its branches was according to Adachi classification^9^: Type I, LGA, CHA, and SA all originated from the celiac trunk; Type II, LGA originated from AA, CHA and SA originated from the celiac trunk; Type III, LGA from AA, CHA, SA and superior mesenteric artery (SMA) from the celiac trunk; Type IV, CHA, SA, SMA, and LGA all originated from the celiac trunk; Type V, LGA and SA originated from CT, CHA originated from SMA; Type VI, Same as type V, but CHA was behind portal vein (PV). The classification of hepatic artery was according to the Hiatt classification^10^: Type I, normal; Type II, LHA from LGA; Type III, RHA from SMA; Type IV, RHA from SMA, LHA from LGA coexist; Type V, CHA originated from SMA; Type VI, CHA originated from the AA. As for the SA, a study has reported that in the majority of cases (3132 specimens), the SA bifurcated into superior and inferior lobar arteries (83.4%), with trifurcation and quadrifurcation in 11.3% and 2.7%, respectively. Five or more lobar branches (1.4%) and a single lobar artery (1.2%) were rarely identified^11^. In terms of surgery, the more complicated the SA branches, the more challenging it is to dissect lymph nodes in the superior pancreas and splenic hilum region. The surgical risk and difficulty will increase accordingly. In this study, we established a new Classification of risk factors for SA combining vascular anatomy and clinical practice. We classified according to whether there is a serious tortuosity or even helix in the trunk of the SA, the number of SA branches, and whether the superior pole branch of SA originates from the middle segment of the SA trunk.

### Imaging study and surgical study

CT was performed using Philips Brilliance 256-slice Helical CT Scanner (Philips Medical Systems, Amsterdam, Netherlands). All patients were forbidden to eat for eight hours before the examination. Iopromide 300 was the contrast agent. The contrast-enhanced CT examination was set to cover the area ranging from the dome of the liver to the lower border of the pelvic cavity. During the enhancement scan, a high-pressure syringe was used to inject the contrast agent through the cubital vein at a rate of 5 ml/s.

In the CTA group, the volume data during patients' arterial enhanced CT phase was transmitted to syngo.via workstation. Then we used volume rendering technology to prepare 3d images of the arteries by selecting CT values suitable for specific target areas and color processing the photos. The above 3d images were fused to obtain the CTA image^12^.

In the ICG group, patients were marked with ICG solution under gastroscope within 16–24 h before surgery. Injection site: four quadrants of the oral side, anal side, the lesser curvature of the stomach, and the greater curvature of the stomach were selected for submucosal injection at the junction of the tumor edge. 1.5 ml of indocyanine green (indocyanine green for injection, 25 mg/piece, diluted to 1.25 mg/ml with sterile water for injection) is injected into the submucosal layer of each point.

All patients underwent laparoscopic radical gastrectomy (D2 lymph node dissection). Complete omentectomy, omental sac resection, perigastric dissection, ligation and severing of perigastric vessels, and lymph node dissection under laparoscopy.

In the CTA group, preoperative 3D CT angiography was used to assist the operator in finding the perigastric arteries, correctly judging the course of the perigastric arteries to guide the lymph node dissection during surgery. In the non-CTA group, routine lymph node dissection was performed.

The ICG group used near-infrared laparoscopy (Stryker company Pinpoint fluorescence laparoscope) to visualize the perigastric lymph nodes during the operation. During the process of lymph node dissection, the natural light mode and the fluorescence mode were switched according to the surgeon's request. ICG labeled surgery aims to remove lymph nodes that are difficult to identify with the naked eye but can be visualized in fluorescence mode. The standard D2 lymph node dissection was performed in the non-CTA group.

### Clinical results

(1) Basic characteristic data: age, sex, and body mass index (BMI); (2) Observed and recorded indicators during surgery: operative time, intraoperative estimated blood loss, and incidence of vascular injury; (3) Postoperative short-term indicators: postoperative first exhaust time, first drinking time, first liquid diet time, postoperative gastric tube and abdominal drainage tube extraction time, postoperative liver function (ALT, AST) and postoperative hospital stay time; (4) Postoperative complications: only grade ≥ II postoperative complications were counted according to clavien-Dindo grading^13^. Complications were defined as adverse events occurring within 30 days after surgery, and only the most severe complications were considered when multiple complications occurred in the same patient; (5) Postoperative pathological results: tumor stage (AJCC), tumor staging was carried out according to AJCC grading criteria^6^. Tumor differentiation, number of total lymph nodes dissected, and number of positive lymph nodes dissected.

### Statistical analysis

SPSS25.0 statistical software was used to analyze the data. We performed propensity score matching (PSM) with all basic characteristic data. A Nearest-neighbor matching was used. The size of the caliper was set as 1:1. We excluded the patients who were outside the caliper or unmatched. The measurement data conformed to normal distribution were expressed using the mean ± standard deviation. Otherwise, the skewed data with the heterogeneity of variance were expressed as median and inter-quartile range. Independent sample T-test or Non-parametric test was used for measurement data, and chi-square test was used for all categorical data. A *P* value < 0.05 was considered significant.

### Conflicts

The authors declare no competing financial interests. The study was approved by the Medical ethics committee of Qilu Hospital of Shandong University (no. 2018-223)and was performed in accordance with relevant guidelines and regulations. All participants provided written informed consent.

## Results

### Basic characteristic data of gastric cancer patients

There were 214 cases in all, 125 cases in the CTA group, and 89 cases in the non-CTA group. There were significant differences between the two groups in surgical approach, surgical procedure, gastrointestinal reconstruction methods, and Charlson Comorbidity Index. After propensity score matching, 75 cases in the CTA group 75 cases in the non-CTA group. There was no significant difference in sex, age, BMI, surgical approach, surgical procedure, tumor stage, tumor differentiation, gastrointestinal reconstruction methods, and Charlson Comorbidity Index between the CTA group and non-CTA group in basic data of patients (*P* > 0.05) (Table 1).

**Table 1:** Basic characteristic data of total gastric cancer patients and gastric cancer patients after PSM.

	Entire cohort			Cohort after PSM		
	CTA (n = 125)	Non-CTA (n = 89)	*P*	CTA (n = 75)	Non-CTA (n = 75)	*P*
**Sex**						
Male	97 (77.6%)	72 (80.9%)	0.559	57 (76.0%)	60 (80.0%)	0.554
Female	28 (22.4%)	17 (19.1%)		18 (24.0%)	15 (20.0%)	
Age (years)	59.34 ± 10.23	60.92 ± 9.34	0.374	60.43 ± 10.27	60.21 ± 9.56	0.712
BMI (kg/cm^2^)	24.15 ± 3.28	24.41 ± 3.63	0.315	24.04 ± 3.30	24.25 ± 3.63	0.515
**Surgical procedure**						
Proximal gastrectomy	12 (9.6%)	13 (14.6%)	0.042	7 (9.3%)	11 (14.7%)	0.346
Distal gastrectomy	68 (54.4%)	33 (37.1%)		36 (48%)	28 (37.3%)	
Total gastrectomy	45 (36.0%)	43 (48.3%)		32 (42.7%)	36 (48.0%)	
**Surgical approach**						
Laparoscopic assistance	83 (66.4%)	71 (79.8%)	0.032	59 (78.7%)	61 (81.3%)	0.683
Total laparoscopic	42 (33.6%)	18 (20.2%)		16 (21.3%)	14 (18.7%)	
**Tumor stage**						
I	52 (41.6%)	34 (38.2%)	0.670	33 (44.0%)	26 (34.7%)	0.247
II	35 (28.0%)	30 (33.7%)		17 (22.7%)	26 (34.7%)	
III	38 (30.4%)	25 (28.1%)		25 (33.3%)	23 (30.7%)	
**Tumor differentiation**						
Low	70 (56.0%)	42 (47.2%)	0.561	39 (52.0%)	36 (48.0%)	0.547
Low–middle	23 (18.4%)	18 (20.2%)		13 (17.3%)	16 (21.3%)	
Middle	17 (13.6%)	19 (21.3%)		11 (14.7%)	16 (21.3%)	
Middle–high	9 (7.2%)	5 (5.6%)		7 (9.3%)	3 (4.0%)	
High	6 (4.8%)	5 (5.6%)		5 (6.7%)	4 (5.3%)	
**Gastrointestinal reconstruction methods**						
Tubular gastric anastomosis	5 (4.0%)	5 (5.6%)	0.025	1 (1.3%)	5 (6.7%)	0.775
Billroth-II	65 (52.0%)	30 (33.7%)		32 (42.7%)	28 (37.3%)	
Roux-en-y	49 (39.2%)	40 (44.9%)		36 (48.0%)	36 (48.0%)	
Reverse puncture anastomosis	5 (4.0%)	8 (9.0%)		5 (6.7%)	6 (8.0%)	
Overlap anastomosis	1 (0.8%)	3 (3.4%)		1 (1.3%)	0 (0%)	
Billroth-I	0 (0%)	3 (3.4%)		0 (0%)	0 (0%)	
**Charlson comorbidity index**						
2	26 (20.8%)	6 (6.7%)	0.030	9 (12.0%)	5 (6.7%)	0.633
3	33 (26.4%)	17 (19.1%)		18 (24.0%)	16 (21.3%)	
4	37 (29.6%)	36 (40.4%)		27 (36.0%)	31 (41.3%)	
5	22 (17.6%)	23 (25.8%)		14 (18.7%)	17 (22.7%)	
6	3 (2.4%)	5 (5.6%)		3 (4.0%)	5 (6.7%)	
7	3 (2.4%)	1 (0.8%)		3 (4.0%)	1 (1.3%)	
8	0 (0%)	1 (0.8%)		0 (0%)	0 (0%)	
10	1 (0.8%)	0 (0%)		1 (1.3%)	0 (0%)	

### Clinical surgical results

By analyzing patients after PSM, we found that the operation time and estimated blood loss in the CTA group were significantly lower than those in the non-CTA group (*P* < 0.05). But there was no significant difference in total lymph nodes, positive lymph nodes, and vascular injury rate (*P* > 0.05) (Table 2).

**Table 2:** Comparison of clinical surgical results.

	CTA (n = 75)	Non-CTA (n = 75)	*P*
Duration of operation (min)*	240 (217.5–270)	270 (235–300)	0.010
Estimated blood loss (ml)*	30 (20–50)	50 (20–50)	0.008
Total lymph nodes*	26 (22–36)	26 (20–39)	0.772
Positive lymph nodes*	0 (0–5)	0 (0–4)	0.975
Vascular injury	1 (0.80%)	2 (2.25%)	1.000

*Data were shown as median and inter-quartile range.

### Short-term recovery situation after the operation

The first exhaust time of the CTA group was significantly earlier than that of the non-CTA group (*P* < 0.05). But there was no significant difference in postoperative complications (≥ II grade), postoperative hospital stay, postoperative drinking water time, postoperative first fluid diet time, postoperative removal of drainage tube time, postoperative liver function (*P* > 0.05)(Table 3).

**Table 3:** Comparison of postoperative short-term recovery.

	CTA (n = 75)	Non-CTA (n = 75)	*P*
Postoperative complications (≥ II grade)	12 (16.0%)	10 (13.33%)	0.644
Postoperative hospital stay (days)	11 (9.75–13)	10 (10–12)	0.680
First drinking water time (days)	5 (4–7)	6 (4–7)	0.453
First fluid diet time (days)	7 (4.75–8)	7 (0–9)	0.646
First exhaust time (days)	5 (4–6)	6 (5–7)	0.011
Gastric tube extubation time (days)	4 (3–6.25)	4 (3–6)	0.594
Left abdominal drainage time (days)	7 (6–9)	7 (6–8)	0.563
Right abdominal drainage time (days)	9 (8–10)	9 (8–10)	0.626
Postoperative liver function (AST)	40 (33–54.5)	45 (32–63)	0.392
Postoperative liver function (ALT)	44 (33–64.75)	40 (30–59)	0.541

### Complications

In this study, a total of 22 patients developed postoperative complications, including 12 in the CTA group and 10 in the non-CTA group, which has no statistically significant difference (*P* > 0.05). Vascular injury is a serious complication during surgery. Two cases occurred in the non-CTA group, one in the CTA group (*P* > 0.05). Two cases of vascular injury in the non-CTA group: intraoperative bleeding was caused by accidental injury during lymph node dissection because the SA is severely tortuous: the upper pole branch of the SA was sent out from the middle part of the SA, which was ligated and severed because we thought it could be the posterior gastric artery. A case of vascular injury in the CTA group: the trunk of SA was damaged because old equipment (electrical coagulation leakage) was discharged at an abnormal site and broke the SA. The patient was transferred to laparotomy for vascular repair and hemostasis.

### Subgroup analysis of ICG in patients with CTA

**Figure 2:**
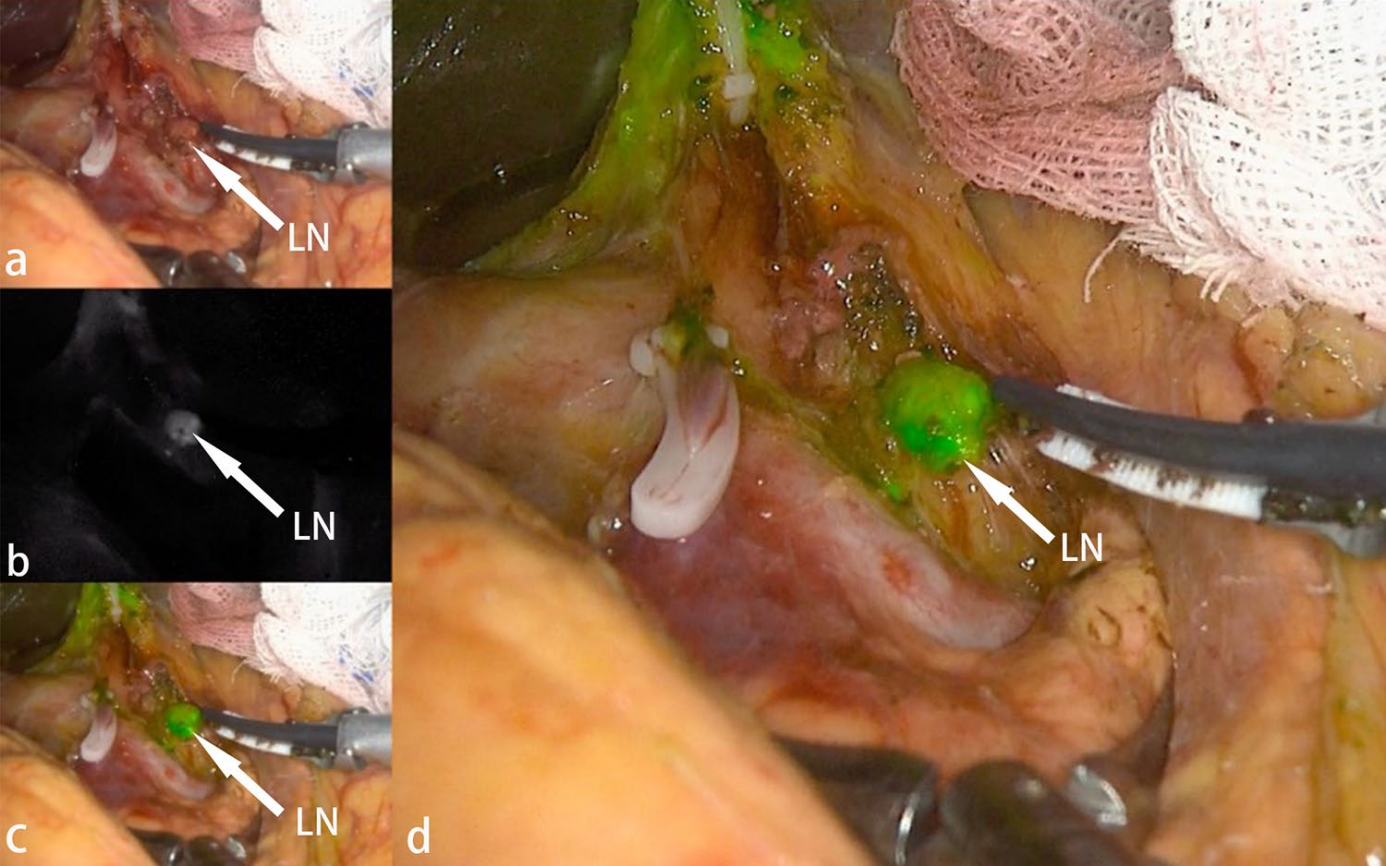
Lymph node images under different modes of laparoscopy with fluorescent labeling of indocyanine green. (**a**) Lymph nodes in normal mode. (**b**) Lymph nodes in contrast mode can more easily identify deeply stained lymph nodes. (**c**,**d**)Green-stained lymph nodes under ICG-labeled fluorescence mode.

In order to evaluate the intraoperative indicators of preoperative CTA combined with ICG, we divided the CTA group into two subgroups by whether or not ICG-labeled fluorescence laparoscopy was performed. Under different modes of fluorescence laparoscopy, we can see that the lymph nodes are presented in the normal state, green stained state, and enhanced white state (Fig. 2).

There were 125 cases in the CTA group, 76 cases in the ICG group, and 49 cases in the non-ICG group. There were significant differences between the two groups in surgical approach, surgical procedure, and tumor stage. After propensity score matching, There were 36 cases in the ICG group, 36 cases in the non-ICG group, and there was no significant difference in basic data of gastric cancer patients (*P* > 0.05) (Table 1).

In our study, the estimated blood loss of the ICG group was significantly less than the non-ICG group (*P* < 0.05). The operation time, the total number of lymph nodes dissected, the total number of positive lymph nodes were not significantly different between the two groups (*P* > 0.05) (Table 4).

**Table 4:** Comparison of intraoperative indicators between ICG group and non-ICG group.

	ICG (n = 36)	Non-ICG (n = 36)	*P*
Estimated blood loss (ml)	35 (22.5–50)	50 (30–80)	0.004
Operation time (min)	240 (212.5–270)	240 (210–295)	0.892
Total lymph nodes	26.5 (20–34.5)	30 (24–42.75)	0.062
Positive lymph nodes	0 (0–5.25)	1 (0–5.75)	0.113

### Branching classification of the celiac trunk and the hepatic artery

**Figure 3:**
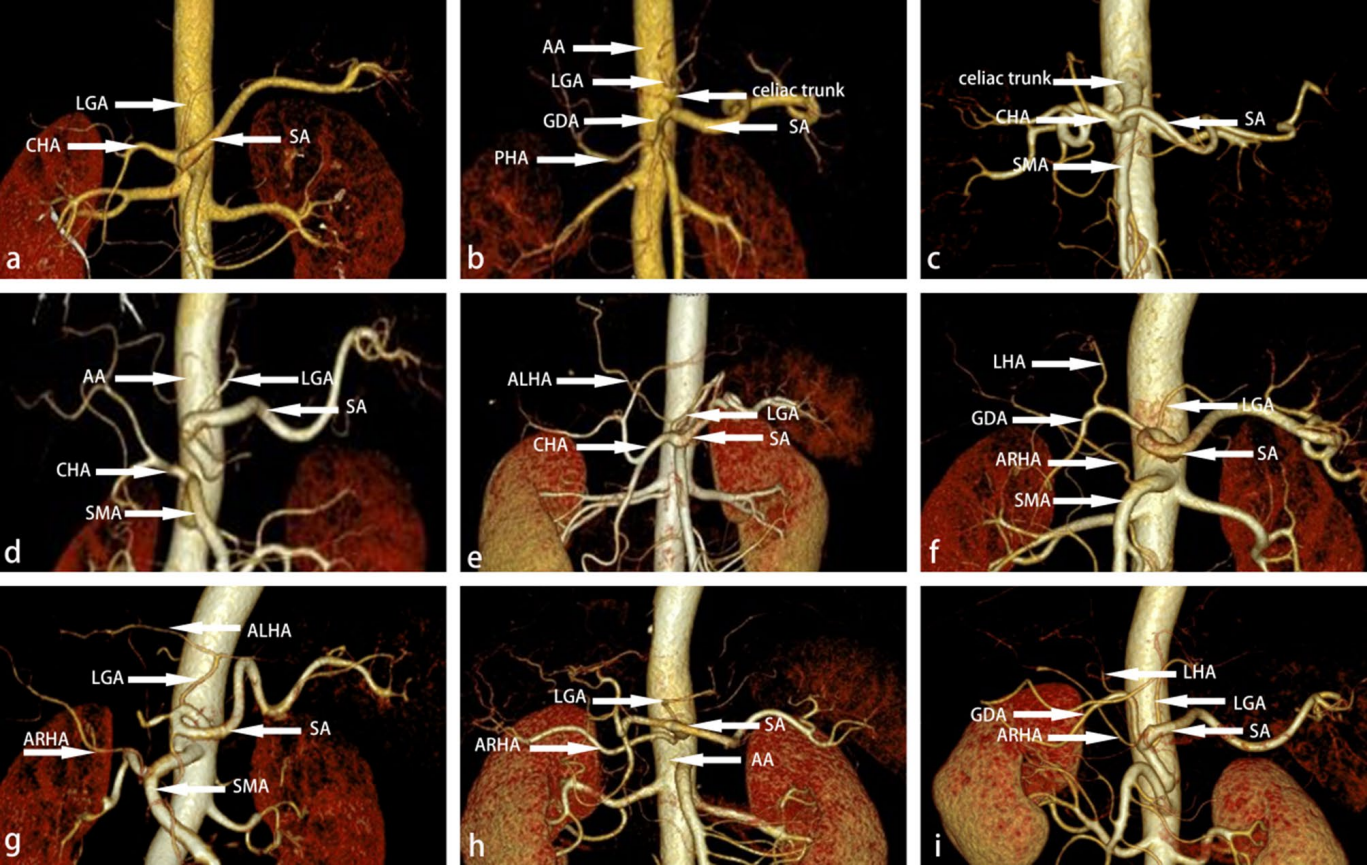
Classification of the celiac trunk. (**a**) LGA, CHA, and SA all originated from the celiac trunk. (**b**) LGA originated from AA, CHA and SA originated from the celiac trunk. (**c**) CHA, SA, SMA, and LGA originated from the celiac trunk. (**d**) CHA originated from SMA. (**e**) ALHA from LGA. (**f**) The ARHA originated from SMA. (**g**) ARHA from SMA, ALHA from LGA coexist. (**h**) In this case, ARHA from AA. (**i**) In this case, ARHA from the celiac trunk.

The celiac trunk branches classification: Type I (118/125, 94.4%), Type II (3/125, 2.4%), Type IV (1/125, 0.8%), Type V (2/125, 1.6%), Type VI (1/125, 0.8%) (Table 5; Fig. 3).

**Table 5:** Branch classification of the celiac trunk.

Classification	Adachi classification		This study	
I	221/252	87.7%	117/125	94.4%
II	16/252	6.4%	3/125	2.4%
III	3/252	1.2%	0/125	0%
IV	6/252	2.4%	1/125	0.8%
V	1/252	0.4%	2/125	1.6%
VI	5/252	2.0%	1/125	0.8%

The hepatic artery classification: Type I (102/125, 81.6%), Type II (9/125, 7.2%), Type III (6/125, 4.8%), Type IV (2/125, 1.6%), Type V (3/125, 2.4%), Type VI (0, 0%), Others: 3 cases (3/125, 1.6%). In one case, the RHA originated from the AA and the PHA originated from the celiac trunk artery. In two cases, RHA from AA (Table 6; Fig. 3).

**Table 6:** Branch classification of hepatic artery.

Classification	Hiatt classification		This study	
I	757/1000	75.70%	102/125	81.6%
II	97/1000	9.70%	9/125	7.2%
III	106/1000	10.60%	6/125	4.8%
IV	23/1000	2.30%	2/125	1.6%
V	15/1000	1.50%	3/125	2.4%
VI	2/1000	0.20%	0/125	0%
Others	0/1000	0.00%	3/125	2.4%

### A new classification of surgical risk factors for SA

**Figure 4:**
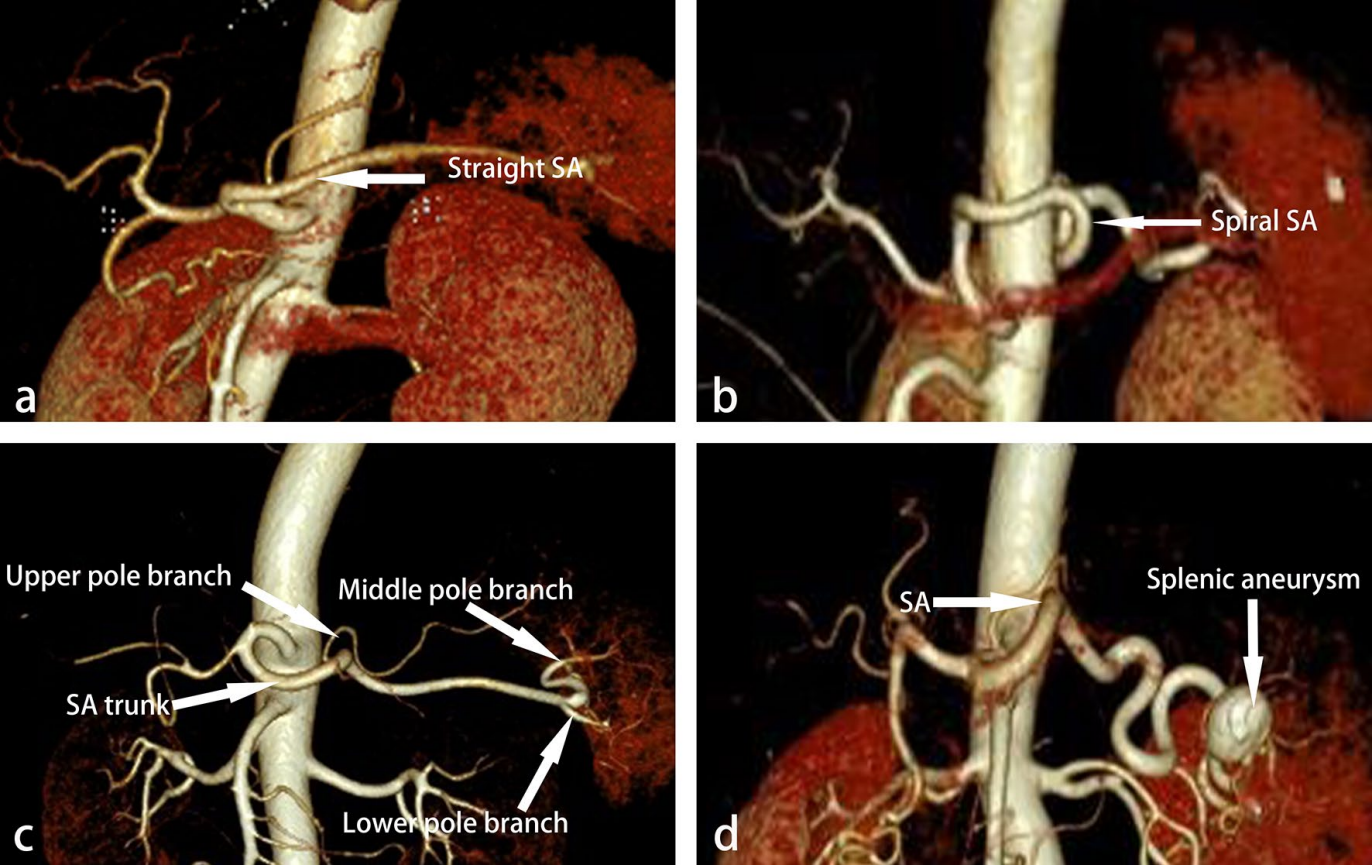
Several anatomical risks of SA. (**a**) A straight SA. (**b**) A spiral SA, as a risk factor for surgery, could significantly increase the rate of vascular injuries. (**c**) The splenic artery is of three-branched type. The middle and lower branches originate, but the upper pole branches originate from the middle parts of the splenic artery. (**d**) In this case, a huge splenic aneurysm was found in the superior branch of the splenic artery.

This study established a new classification of surgical risk factors for SA combining vascular anatomy and clinical practice. We classified according to whether there is a serious tortuosity or even helix in the trunk of the SA, the number of SA branches, and whether the superior pole branch of SA originates from the middle segment of the SA trunk (Fig. 4). In addition, there was 1 case of splenic aneurysm found in the CTA examination (Fig. 4), which was removed in operation.

The SA is classified into ten types: The severe risk type: IIa(1.6%), IIIa(5.6%). This type is characterized by all three surgical risk factors: seriously tortuous SA, two or three branches, and the superior pole branch of SA originates from the middle segment of the SA trunk, which is easy to cause wrong judgment of the operation and bring a high risk to surgery. The moderate risk type: IIb(35.2%), IIIb(10.4%), Va(0.8%), VIa(7.2%). Two surgical risk factors characterize this type. The rest were the types with low risk of surgery: I(1.6%), IV(0.8%), Vb(28.0%), VIb(8.8%) (Table 7; Fig. 4).

## Discussion

Recently, laparoscopic radical gastrectomy has been widely used in the treatment of gastric cancer and achieved good therapeutic effects^14^. However, enhanced CT and other preoperative examinations cannot accurately judge the anatomy of essential vessels, thus affecting lymph node dissection, which has always been an urgent problem to be solved in laparoscopic surgery. In this study, preoperative CTA was used to guide the operation by helping the operator understand the anatomical characteristics of the artery before operation. The results showed that the operative time and estimated blood loss in The CTA group were significantly lower than those in the non-CTA group (*P* < 0.05) (Table 2). And the first exhaust time of the CTA group was significantly earlier than that of the non-CTA group (*P* < 0.05), but there were no significant differences in other clinical outcomes between the two groups (*P* > 0.05) (Table 3). For subgroup analysis, except the estimated blood loss of the ICG group was significantly lower than the non-ICG group (*P* < 0.05), there was no significant difference in other clinical surgical results (*P* > 0.05) (Table 4).

A large number of studies had shown^5, 15–17^ that preoperative CTA examination can reduce the amount of blood loss, shorten the operation time and even improve the detection rate of lymph nodes in laparoscopic radical gastrectomy. In our study, the estimated blood loss and operation time was lower than that in the group of CTA (*P* < 0.05). We considered that CTA could help us understand the course characteristics of perigastric arteries, to shorten the time to find and identify perigastric vessels. More accurate identification of blood vessels will reduce intraoperative bleeding undoubtedly. There was no significant difference in the total number of lymph nodes and the number of positive lymph nodes between the CTA group and the non-CTA group (*P* > 0.05), which could be related to the veteran experience of the surgeon and careful dissection.

The first exhaust time of the CTA group was significantly earlier than that of the non-CTA group (*P* < 0.05). Other postoperative short-term recovery indicators between the two groups was no apparent difference (*P* > 0.05). We believe that the operation under the guidance of preoperative CTA may save operation time and thus reduce the time for patients to endure pneumoperitoneum. As is known to all, long time to stay pneumoperitoneum will affect the recovery of patients' gastrointestinal function after an operation, which is expressed as the first exhaust time. Additionally, there was no significant difference in the injury of vessels in this study (*P* > 0.05). However, our data showed that there was only 1 case of SA injury because of device reason (coagulation hook damage) in the CTA group, which was an unexpected accident. There was no significant statistical significance in perigastric vascular injuries, which may be caused by a small sample size.

Notably, in the CTA group, the ICG group had less blood loss than the group without ICG (*P* < 0.05), but there was no significant statistical difference in the operation time, number of total lymph nodes and positive lymph nodes. So we found that preoperative CTA combined with ICG labeled was more effective in reducing blood loss. We considered that preoperative CTA and ICG labeled could effectively distinguish small blood vessels from lymphatic vessels or lymph nodes by fluorescence mode, effectively avoiding or reducing bleeding caused by small blood vessel injury and reducing the blood loss. Suitable identification is particularly effective when dissection of the upper pancreas, mainly in reducing iatrogenic damage to the pancreas, thereby reducing the incidence of postoperative pancreatic fistula. In addition, we expected that the time to carry out the fluorescence pattern recognition process would prolong the total operation time. There was no significant difference in the operation time between the two groups (*P* > 0.05), indicating that the ICG labeled fluorescence laparoscopy did not extend the operation time.

For decades, the classification of perigastric arteries has been a concern by gastrointestinal surgeons. Many studies^9, 10, 18^ reported the classification of gastric peripheral arteries and detailed classification. Among them, Adachi classification^9^ and Hiatt classification^10^ are more classical. We conducted vascular classification according to these two classifications, discussed vascular variation to avoid accidental injury caused by vascular variation, and established a new classification of risk factors for SA, designed to make surgery safer.

**Figure 5:**
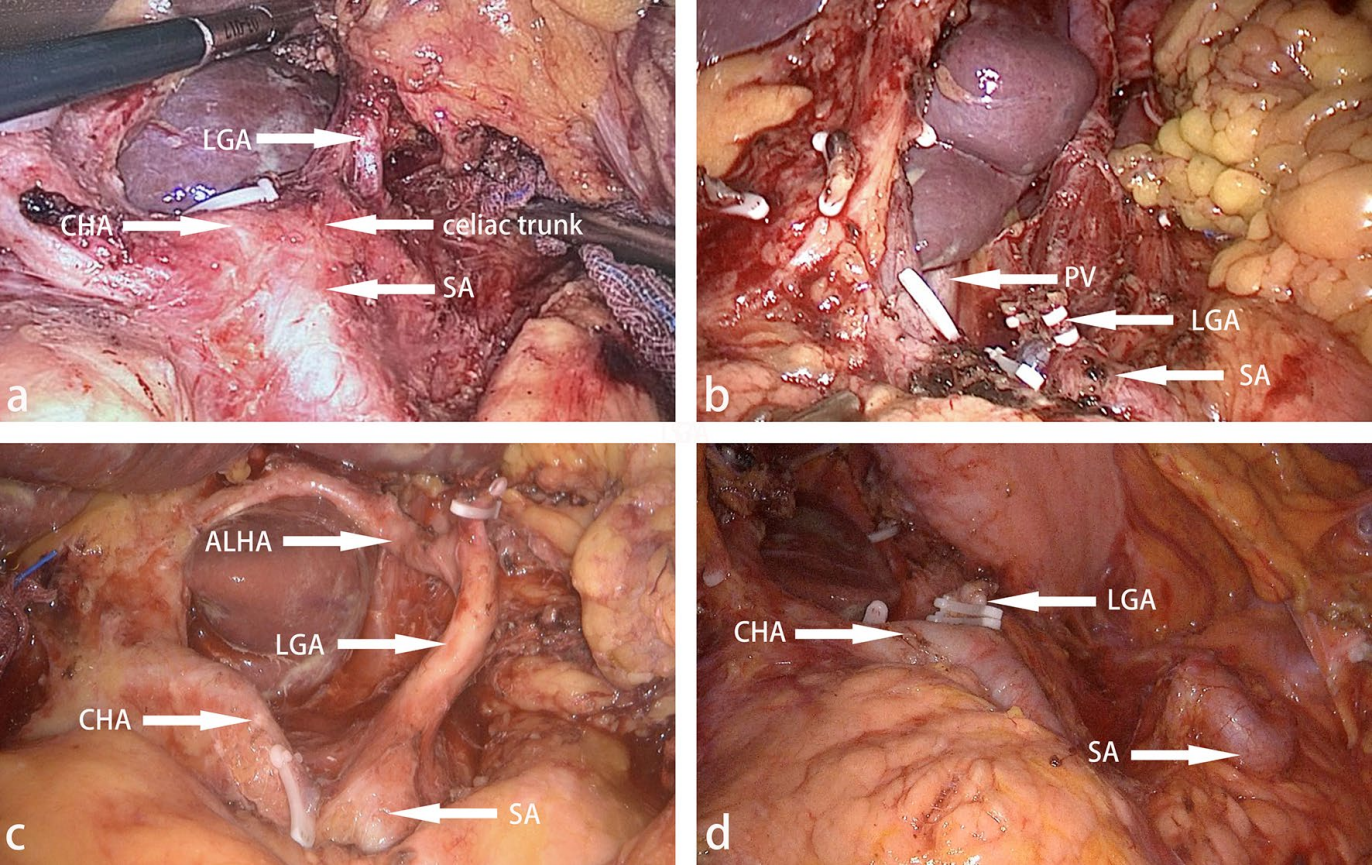
Several typical vascular variations during surgery. (**a**) LGA originated from AA, CHA and SA originated from the celiac trunk. (**b**) CHA originated from SMA. So we can't find CHA in the suprapancreatic region, and the portal vein is directly exposed in the surgical field without occlusion of the CHA. (**c**) ALHA from LGA. (**d**) A spiral SA.

Some studies have shown that LGA directly derived from AA accounted for 2.3%^15^. There were 3 cases of type II variations (2.4%) in our study (Figs. 3 and 5), the same as the study above. Knowing this, we could avoid the risk of mistakenly severing the splenic artery. In Marco's study^19^, a complete tetrafurcated trunk was detected in 4/596 CTs (0.7 %). In this research, there was only one case of type IV variation (0.8%): CHA, SA, SMA, and LGA were all originated from CT. In this variation, attention should be paid to avoid misjudgment of blood vessels. Keishi 's research^20^ statistics showed that 28 of 714 cases (3.9%) CHA originated from SMA. In our study, CHA originated from SMA in 3 patients (2.4%) (Figs. 3 and 5). When we dissect the lymph nodes of Group 8a, the portal vein is not covered by the CHA, which is a marker vessel, and will be directly exposed to the surgical field^20^. If not foreseen before surgery, the risk of portal vein injury will undoubtedly increase. The total variation rate of the hepatic artery in our study was 17.6%, which was similar to Hiatt's study (24.3%)^10^. However, the variation of the hepatic artery is complex and diverse, and the Hiatt classification^10^ cannot cover all variations^21^. In this study, we found two types of variation, in which abnormal RHA provides the main blood supply to the right liver lobe. These two variations were not described in Hiatt classification^10^: in 1 case, the RHA originated from the celiac trunk; in 2 cases, RHA originated from AA (Fig. 3).

According to relevant reports^22^, 15–20%ALHA may originate from The LGA, which is sometimes the only arterial blood flow in the left hepatic lobe. ALHA can be divided into alternative LHA or accessory LHA, which is difficult to distinguish the two conditions during surgery. There were 9 cases of type II variation in this study: abnormal LHA from the LGA (Figs. 3 and 5). Research reports that preoperative CTA combined with thin layer scanning at arterial phase could well differentiate these two^23^. Indeed, CTA can help us identify whether there is an abnormal LHA and determine whether it is the only blood supply to the liver, which will provide predictive information for the operator's intraoperative decision-making. Interestingly.

In 125 cases, the detection rate of the RGA was 65.6% (82/125). RGA is usually thin and limited by imaging technology, CTA sometimes can not display the course of RGA^23^, and the detection rate was not high. But there is no denying the importance of identifying the RGA. If the RGA is parallel or close to the PHA, we need to be cautious. We may need to ligate and cut off the RGA far from its root. Because without preoperative CTA evaluation, a careless operation may lead to the wrong ligation of PHA, which will lead to liver ischemia.

The abnormal SA was classified according to three risk factors that affect surgery: the number of SA branches, whether the SA trunk was tortuous, and whether the superior pole branch of SA originates from the middle segment of the SA trunk (Table 7). Preoperative understanding of the bending degree of SA would be beneficial to complete dissection of No.11 lymph node and reduce the risk of SA injury. In one case of the non-CTA group, a superior pole of the splenic artery, which originated from the middle segment of the splenic artery, was mistaken for the short gastric artery and ligated, resulting in insufficient blood supply to the remnant stomach and ischemia of the superior pole of the spleen. In addition, we found that tortuous SA also brought a high risk of surgery. If the splenic artery is tortuous(Figs. 4 and 5), sometimes the tortuous splenic artery is mistakenly dissected as a lymph node, leading to serious vascular damage. In addition, 1 case of the giant splenic aneurysm was found by preoperative CTA examination, which was of high surgical risk (Fig. 4). It has been reported that laparoscopic treatment of splenic aneurysms is a safe, effective, and minimally invasive option^24^. We made reasonable surgical strategies to remove the splenic aneurysm during radical gastrectomy.

**Table 7:** Splenic artery classification.

The course of the SA trunk	Our study	Proportion (n%)	Number of branches	Whether the superior pole branch of SA originates from the middle segment of the SA trunk	Our study	Proportion (n%)	Type
Seriously tortuous	68	54.4	One branch	–	2	1.6	I
			Two branches Two branches	Yes	2	1.6	IIa
				No	44	35.2	IIb
			Three branches Three branches	Yes	7	5.6	IIIa
				No	13	10.4	IIIb
Straight	58	46.4	One branch	–	1	0.8	IV
			Two branches Two branches	Yes	1	0.8	Va
				No	35	28.0	Vb
			Three branches Three branches	Yes	9	7.2	VIa
				No	11	8.8	VIb

Literature on the laparoscopic-assisted gastrectomy learning curve suggests that surgeons become proficient after 40 operations^25^. So we have reason to believe that preoperative CTA will play a significant role in safely and efficiently completing surgery for surgeons still overcoming the learning curve.

There were still several limitations. The sample size of this study is small, and this research is a retrospective analysis, not enough to explain the problem. So, It is necessary to conduct prospective studies and expand the sample size based on this study. Since the inferior pyloric artery, right gastroepiploic arteria, and left gastroepiploic arteria are thin, 3d-CT reconstruction technology has a limited ability to develop them, so this study did not analyze them. In the future, more precise angiography technology could be used to identify them, and their course characteristics can be analyzed.

According to the literature^26^ and our study results, it had been confirmed that preoperative CTA is helpful in laparoscopic operation. Preoperative CTA could objectively evaluate the course and variation of perigastric arteries, helping us identify blood vessels, thereby shortening the operation time and reducing estimated blood loss. ICG labeling could further reduce the amount of blood loss and the risk of iatrogenic injury. Preoperative CTA is expected to be widely used clinically in combination with Indocyanine green-labeled fluorescence laparoscopy.

## Conclusion

According to the literature and our study results, preoperative CTA could objectively evaluate patients' vascular route and variation and then help us avoid or decrease the risk of vascular injury and bleeding. When combined with ICG labeled fluorescence laparoscopy, it could further reduce the risk of iatrogenic injury during the operation and improve postoperative recovery.
